# Livedoid vasculopathy: A review with focus on terminology and pathogenesis

**DOI:** 10.1177/1358863X221130380

**Published:** 2022-10-26

**Authors:** Harish Eswaran, Paul Googe, Priyanka Vedak, William A Marston, Stephan Moll

**Affiliations:** 1 Department of Medicine, Division of Hematology, University of North Carolina School of Medicine, Chapel Hill, NC, USA; 2 Department of Dermatology, University of North Carolina School of Medicine, Chapel Hill, NC, USA; 3 Department of Surgery, University of North Carolina School of Medicine, Chapel Hill, NC, USA; 4 Blood Research Center, University of North Carolina School of Medicine, Chapel Hill, NC, USA

**Keywords:** livedoid vasculopathy, skin, thrombosis, thrombotic vasculopathy, wound/ulcer

## Abstract

Livedoid vasculopathy (LV) is a rare thrombotic vasculopathy of the dermis characterized by painful, relapsing ulcers over the lower extremities. Diagnosis is challenging due to the overlap in clinical appearance and nomenclature with other skin disorders. Treatment selection is complicated by poor understanding of the pathogenesis of LV and lack of robust clinical trials evaluating therapy efficacy. The terminology and pathophysiology of LV are reviewed here, along with its epidemiology, clinical and histologic features, and treatment options. A diagnostic pathway is suggested to guide providers in evaluating for comorbidities, referring to appropriate specialists, and choosing from the available classes of therapy.

## Introduction

**Figure 1.**
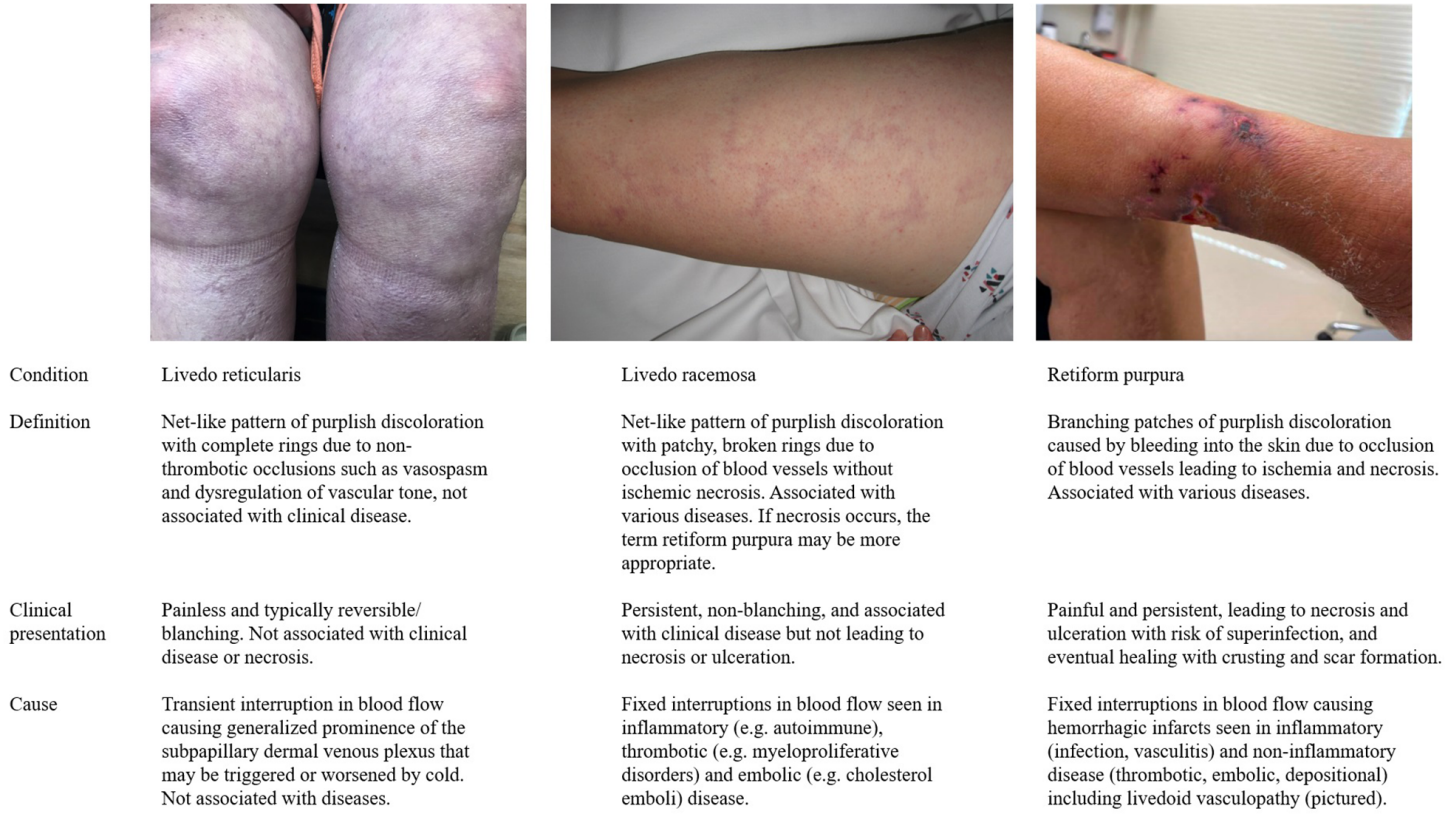
Definitions, clarification of terminology, and clinical appearance of reticular skin eruptions of vascular etiology.

Livedoid vasculopathy (LV) is a debilitating condition characterized by thrombosis of dermal vessels without significant inflammatory infiltrate leading to recurrent, painful ulcers of the lower extremities. Vascular medicine and hematology specialists may be asked to comment on possible etiology, further diagnostic workup, and treatment options in patients with ulcerating skin lesions or thrombotic vasculopathy on skin biopsy. A good understanding of the various terminologies used by dermatology to describe the manifestations of cutaneous vascular disease is essential in the initial assessment of the patient. Figure 1 defines these terms and lists the key clinical findings, etiologies, and associated conditions.

Diagnosis and treatment of LV can be challenging for providers. Although the classic clinical and histological features of LV have been well described, many of these features overlap with findings in other ulcerative skin disorders such as venous stasis. Additionally, the nomenclature of LV can be confusing, the mechanisms leading to microvascular thrombosis are the subject of debate, and optimal treatment selection is unclear.

We review the available literature regarding LV with a focus on its pathogenesis, and recommend the use of more descriptive terminology that reflects the mechanisms that contribute to its development.

## Epidemiology and clinical features

The incidence of LV is estimated to be 1:100,000 per year, and women are predominantly affected at a ratio of 3:1.^1^ Most patients with LV were previously healthy, with no identifiable medical comorbidity.^2^ Mean age of onset is in the 30s, setting up patients for decades of functional impairment.^3–5^ Lack of provider familiarity with LV contributes to significant diagnostic delay, and the median interval between onset of skin lesions and histologic diagnosis is 3.4 years.^4^ We are not aware of any familial cases of LV, but one report suggested there might be ethnic clustering in individuals of Jewish heritage from Georgia.^6^

**Figure 2.**
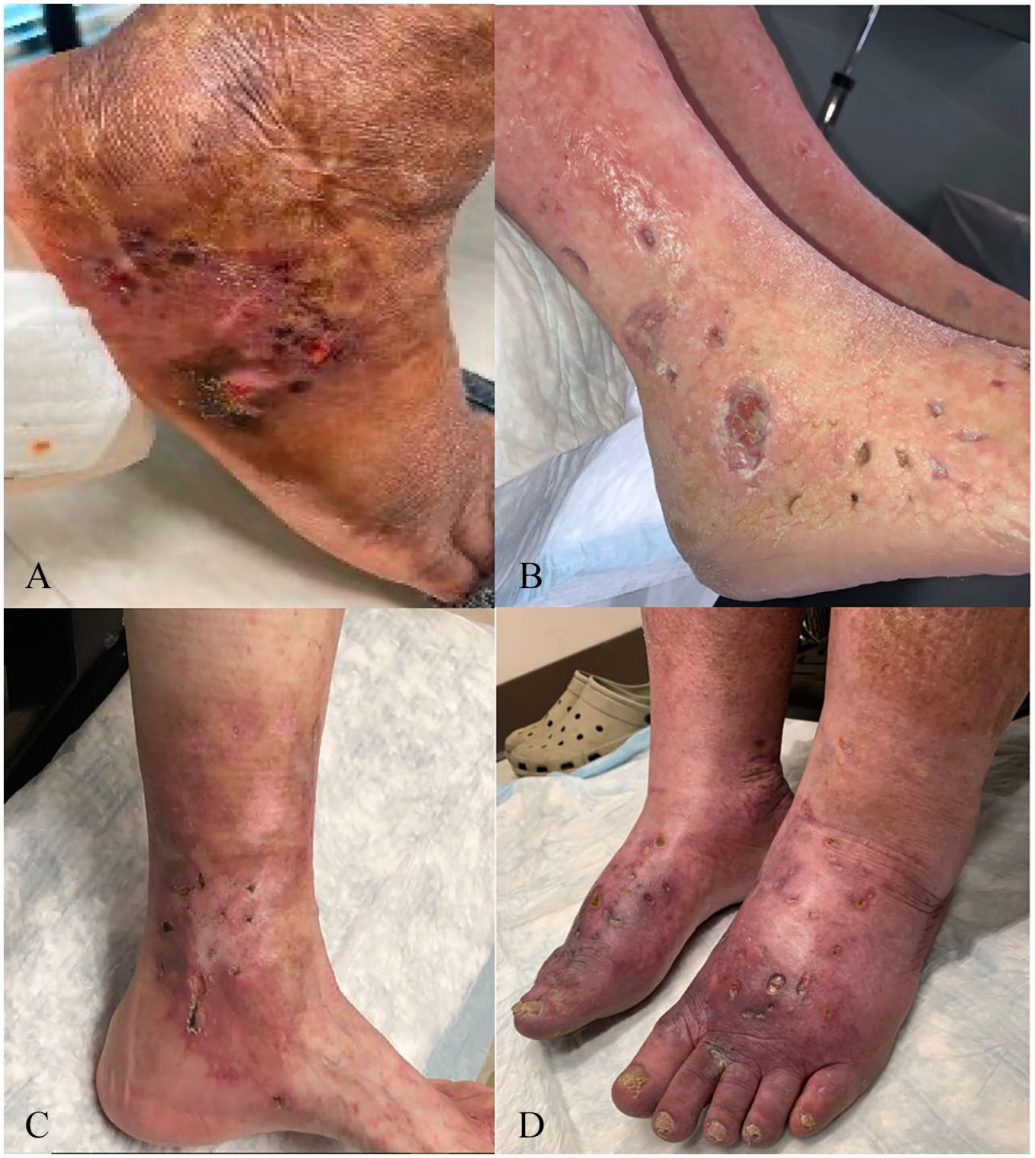
Clinical features of livedoid vasculopathy (LV) skin lesions. **(A)** LV in a patient with sickle cell disease with retiform purpura and ulceration. **(B)** Ulcers in idiopathic LV. Most are sub-centimeter but some have coalesced into larger ulcers. **(C)** LV in a patient with concurrent venous insufficiency and monoclonal gammopathy of undetermined significance. **(D)** Healed ulcers surrounded by areas of atrophie blanche and hyperpigmentation.

LV lesions develop and progress through typical stages (Figure 2). Fixed, violaceous macules and patches appear first, some of which may be stellate in appearance and are referred to as noninflammatory retiform purpura in the dermatology literature (Figure 1). The classic clinical findings are of sub-centimeter ulcers which form within these lesions and are associated with severe pain. These ulcers may take months to heal, eventually forming painless, white, stellate scars known as *atrophie blanche*.^2,3,7^ Lesions are typically bilateral, affecting the ankle, foot, and shin.^4^ The natural history of LV is for lesions to relapse and remit without a clear trigger, and it is common for a patient to exhibit multiple lesions in different stages of healing.^8^ Chronic pain and wound care associated with ulcers present a significant burden to the quality of life of affected patients.^9^

The clinical features of LV are not specific for diagnosis. Livedoid vasculopathy is a potential cause of noninflammatory retiform purpura, but the differential diagnosis includes other causes of microvascular occlusion.^10^ Atrophie blanche is a nonspecific finding that may also be observed as part of the natural healing process of venous stasis ulcers.^11^ The appearance and location of lesions may also vary widely. For example, retiform purpura may rarely be seen on the arms or trunk, and smaller ulcers may coalesce to form larger ones.^1,12^ For these reasons, patients should undergo skin biopsy whenever they present with the classical clinical LV findings, there is an atypical wound that might be associated with LV, or a wound does not respond to usual treatment. Deep, 4–6-mm punch or excisional biopsies at the ulcer margin including surrounding healthy tissue are recommended for diagnosis.^13,14^

## Histopathology

**Figure 3.**
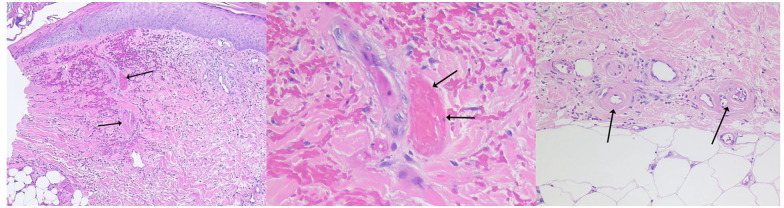
Histopathology of livedoid vasculopathy skin lesions. **Left:** Livedoid vasculopathy in a biopsy of skin shows superficial dermal edema, capillary telangiectasia, extravasation of red blood cells, and intraluminal capillary thrombi (arrows). The thrombosed capillaries do not have inflammation. H&E 10×. **Middle:** Livedoid vasculopathy in a skin biopsy with an occlusive, intraluminal thrombus (arrows) with adjacent ischemic changes in a sweat duct and surrounding hemorrhage. There is no inflammation. H&E 40×. **Right:** Chronic change noted in skin biopsy of livedoid vasculopathy: thickening of capillary walls with pink, glassy (hyalinized) collagen (arrows). There is no inflammation or thrombosis. H&E 20×.

The primary histologic feature of LV is focal thrombosis within the lumina of distal dermal vessels (Figure 3).^15^ Involved vessels are those that are less than 40 μm in diameter that have no internal elastic lamina and very little smooth muscle. Capillaries are predominantly affected, followed by postcapillary venules and arterioles.^16^ Capillaries may also have intramural fibrin deposition with patent lumina. Thrombosis is typically limited to the superficial to mid-dermis, and only occurs rarely in the subcutaneous fat.^17,18^ The epidermis is uninvolved except where there is ulceration, or may be thinned in areas of atrophie blanche.^15,17^ Intervening vessel segments are spared, which may cause findings to be missed on a single biopsy.^14^

The distinction between vasculitis and occlusive vasculopathy is important to appreciate as the absence of vasculitis is pathognomonic for LV. Vasculitis is characterized by inflammatory cells infiltrating and causing damage to vessel walls; leukocytoclasia, or degeneration of neutrophils, may be present, along with fibrinoid necrosis of vessel walls and connective tissue degeneration.^16^ In LV, the structure of vessel walls is largely preserved, though segments of vessel walls associated with thrombosis are thickened, with endothelial cell proliferation, mural fibrin, and subintimal hyalinization. Additionally, there is only minimal lymphocytic inflammatory infiltrate.^15^ However, this does not imply the complete absence of immunoreactants. Direct immunofluorescence studies have shown complement and immunoglobulin (C3>IgM>IgG>IgA) deposition in vessel walls in most patients with LV.^19,20^ Immunoreactant deposition is not specific to LV, and may be found in most inflammatory disorders of the skin.^21^

## Terminology and classification

Livedoid vasculopathy has gone by multiple names, reflecting an evolving understanding of its pathogenesis.^22^ Initially, its name reflected its clinical features. Milian first described atrophie blanche in 1929, but attributed it to sequelae of secondary syphilis or tuberculosis.^23^ Atrophie blanche is now understood to be a feature present in a variety of disorders including LV.^11^ ‘Livedo reticularis with summer ulceration’ was used briefly due to the presence of livedo-like (bluish) skin discoloration in both LV and livedo reticularis, as well as the observation from case series that flares might be triggered by warmer weather.^24^ However, livedo reticularis is painless without ulceration or specific histopathologic correlate and has no clinical untoward effects (Figure 1).^25,26^ Additionally, larger observational studies have shown only a minority of patients with LV report an association of flares with warmer months.^5^ More recently, the term ‘painful purpuric ulcers with reticular pattern of the lower extremities’, was suggested, but was not sufficiently specific to LV either.^27^

In the 1960s, LV began to be defined by its histologic appearance. The terms ‘livedo vasculitis’ and ‘segmental hyalinizing vasculitis’ were used initially due to suspicion that the vascular changes were the result of an inflammatory process.^15^ It was not until the 1980s that ‘livedoid vasculopathy’ emerged to reflect its suspected thrombo-occlusive etiology.^28,29^ Unfortunately, livedoid vasculitis persists in the 2004 revision of the International Classification of Diseases (ICD-10).^30^ Although this has been updated to livedoid vasculopathy in the 2022 ICD-11, livedoid vasculitis can be found throughout current literature and clinical practice.^31,32^

Livedoid vasculopathy may be understood as part of (i.e., a subgroup within) a broad category of cutaneous vascular disorders that appear as branching or net-like purple patches. This umbrella category includes livedo reticularis, livedo racemosa, and retiform purpura (Figure 1). There is discordance in the literature on the exact definitions of these terms, which is compounded by confusion related to their phonetic similarity. However, we recommend strictly distinguishing between them for clarity. Livedo reticularis is a benign condition characterized by net-like lesions caused by clinically insignificant, and often transient, interruptions in blood flow that do not lead to ulceration.^25,26,33^ Livedo racemosa and retiform purpura are fixed eruptions caused by lasting vascular occlusion.^10,26^ Livedo racemosa and retiform purpura may be viewed as a spectrum, with retiform purpura resulting from more severe, widespread, or larger vessel occlusion accompanied by hemorrhagic infarction, ischemic necrosis, and ulceration. In livedo racemosa, collateral circulation may help mitigate ischemia, and lesions are not associated with necrosis or ulceration. Several of the same diseases may cause both livedo racemosa and retiform purpura, though LV is typically associated with the latter.

The irreversible violaceous skin discoloration and necrosis of retiform purpura may be seen in several conditions that must be considered and excluded prior to diagnosis of LV.^16^ Inflammatory causes of retiform purpura include small and medium vessel vasculitis, mixed cryoglobulinemia, calciphylaxis, oxalosis, and angioinvasive infection. These are readily distinguished from LV by the presence of vessel wall damage on biopsy.^10^ Anticoagulant necrosis has a similar histologic appearance to LV with dermal vascular occlusion, but can be distinguished by clinical clues (i.e., anticoagulant use) and the distribution of lesions to areas of the body with increased subcutaneous fat.^34^ The cryopathies (type I cryoglobulinemia, cold agglutinin disease, and cryofibrinogenemia) and monoclonal gammopathies such as Waldenström macroglobulinemia may be diagnosed by characteristic clinical features and the identification of the respective proteins in serum or plasma.^35^ Finally, dermal occlusion caused by microorganism and cholesterol emboli may be identified by the presence of deposits in biopsy specimens.^36,37^

## Pathophysiology

There is limited knowledge regarding the causes of dermal vessel thrombosis in LV, what prompts its first occurrence and triggers its relapses, why it is largely limited to the lower extremities, and why there appears to be a predilection for females. Several reasons may account for our poor understanding of its pathophysiology, including the rarity of the disease contributing to a reliance on case series and confusing terminology leading to inclusion of patients with nonclassical findings in etiological investigation.^22,38^ We examine the varied mechanisms theorized to cause LV below, which are summarized in Table 1.

**Table 1.** Illustrative studies of livedoid vasculopathy pathogenesis.

Mechanistic pathway	Relevant findings	Study	Type	Population
Plasmatic hypercoagulability	Elevated serum levels of fibrinopeptide A	McCalmont et al., 1992^28^	Prospective case series	6 patients with LV and active, new lesions vs controls
Decreasedfibrinolysis	Elevated PAI-1 antigen levels and specific activity	Agirbasli et al., 2011^41^	Prospective case series	20 patients with biopsy-proven LV vs controls
	Increased PAI-1 mRNA expression in paraffin blocks	Agirbasli et al., 2017^73^	Retrospective case series	14 patients with biopsy-proven LV vs controls
	Increased lipoprotein (a) expression in skin biopsy specimens	Espinel et al., 2017^44^	Prospective case series	38 patients with biopsy-proven LV and active lesions vs 9 healthy controls
	Presence of fibrin cuffs and PAI-1 in biopsy specimens by immunofluorescence	Brakman et al., 1992^74^	Prospective case series	10 patients with atrophie blanche ulcers and 10 healthy controls
Platelet hyperaggregability	Increased expression of platelet P-selectin	Papi et al., 1998^45^	Prospective case series	8 patients with idiopathic LV vs 20 patients with CSVV and 20 controls
T cell activation	Elevated serum IL-2 and soluble IL-2 receptor levels	Papi et al., 1998^45^	Prospective case series	8 patients with idiopathic LV vs 20 controls
Endothelial dysfunction	Decreased flow-mediated vasodilation	Yang et al., 2012^58^	Prospective case series	16 patients with LV and active ulcers vs matched controls
Other	Increased methylene tetrahydrofolate reductase polymorphisms (impairment in homocysteine metabolism)	Lee and Cho, 2021^75^	Retrospective case series	28 patients with biopsy-proven LV vs 69 controls

CSVV, cutaneous small vessel vasculitis; IL, interleukin; LV, livedoid vasculopathy; PAI-1, plasminogen activator inhibitor-1.

The final steps leading to pain and skin findings in LV are clearer.^1^ Transcutaneous oximetry measurements demonstrate reductions in oxygen delivery in most patients with LV, suggesting that oxygen diffusion is impeded by thrombus formation and intramural fibrin deposition.^2^ Pain is the hallmark symptom of LV and is thought to be mediated by microvascular ischemic infarction.^3^ Impairments in the capillary microcirculation are thought to mediate skin discoloration in all forms of livedoid skin changes (Figure 1).^1^

### Hypercoagulability

The presence of widespread microvascular thrombosis suggests that pathologic activation of the coagulation cascade may play a role in the disorder. Although most cases of LV are not associated with identifiable comorbidity, numerous studies have been published suggesting associations between livedoid vasculopathy and thrombophilias such as antiphospholipid syndrome, hyperhomocysteinemia, proteins C, S, and antithrombin deficiency, and Factor V Leiden and prothrombin G20210A gene mutations.^2,8,39,40^ Prospective studies have shown elevations in markers of hypercoagulability such as fibrinopeptide A, lipoprotein (a), and plasminogen activator inhibitor-1 activity.^28,41–44^ Increased P-selectin expression has been found in patients with LV, suggesting that platelet activation may also play a role.^45^ However, in the absence of large and well-designed case–control studies, it is impossible to tell whether observed thrombophilias are more common in patients with livedoid vasculopathy than the general population, coincidental or contributory. Similarly, it is challenging to know whether observed markers of hypercoagulability are a cause or a consequence of the disorder.

Anticoagulation is the most commonly reported treatment for LV, providing further support for its procoagulant pathogenesis. Antiplatelet therapies have demonstrated benefit in some case series, and low-dose tissue plasminogen activator (tPA) has been shown to improve tissue oxygenation and ulcer healing.^38,43^ Sildenafil, a vasodilatory agent, has also been beneficial.^46^

### Contributions of inflammatory pathways

The role of immune dysregulation in LV is controversial.^22,28,47,48^ LV is characterized by only minimal perivascular lymphocytic inflammatory infiltrate without leukocytoclasia, distinguishing it from cutaneous small vessel vasculitis.^1,15^ However, most immunohistopathologic studies of LV show complement and immunoglobin deposition in vessel walls in both primary and secondary forms of the disorder.^2,19,20^ C3 is the most predominant immunoreactant, followed by IgM, IgG, and IgA. Whether the presence of lymphocytes and immunoreactants represents the primary pathologic process or is a secondary reaction to thrombosis is unclear.^47^ However, elevated levels of interleukin (IL)-2 and soluble IL-2 receptor have been found in the serum of patients with LV, suggesting that lymphocyte activation may contribute to the development or propagation of LV.^45^ Additionally, LV is associated with autoimmune disease, such as systemic lupus erythematosus, Sjogren’s syndrome, and rheumatoid arthritis.^3,49^

The efficacy of anabolic steroids and intravenous immunoglobulin (IVIG) as second-line therapies for LV provides further support for an inflammatory pathway hypothesis.^38^ The therapeutic mechanism of IVIG is thought to be related to its elimination of circulating immune complexes and autoantibodies as well as inhibition of complement-mediated damage that may be triggered by activation of the coagulation cascade.^50^ Recently, reduction in pain and healing of ulcers have been described with etanercept (tumor necrosis factor (TNF) inhibitor), tofacitinib (JAK 2 inhibitor), and rituximab (monoclonal antibody against CD20), among other immunomodulatory agents.^51–53^

### Contribution of venous stasis

The distribution of LV lesions limited to the lower extremities suggests that stasis and increased hydrostatic pressure in the dermal microcirculation may contribute to thrombosis.^22^ It is important to note that chronic venous insufficiency can itself produce painful lower-extremity ulcers, and some authors recommend that patients be ruled out for venous insufficiency prior to making a diagnosis of LV.^1^ However, several studies have found that venous stasis and LV may coexist and can be distinguished by idiosyncratic clinical and histologic features.^2,14^ Ulcers on the bilateral malleoli and dorsum of the foot are described in LV, whereas venous stasis is associated with peripheral edema and large, shallow ulcers predominantly on the medial distal leg.^54^ The basic histopathology of venous hypertension includes superficial dermal capillary proliferation, dermal fibrosis, hemosiderin deposition, and thickening of the walls of small and medium-sized blood vessels.^55^

Two recent studies have found that in small groups of patients with livedoid vasculopathy and superficial venous reflux, ablation of affected vessels resulted in improvement of pain and resolution of ulcers.^56,57^ The findings led Chow et al.^57^ to describe livedoid vasculopathy and venous stasis as a ‘continuum’, though it is unclear whether patients met stringent histologic criteria for LV. More research is needed regarding the relationship of LV and venous stasis and whether conservative measures such as compression therapy have benefit in idiopathic LV.

### Other mechanisms

Livedoid vasculopathy has been described in association with a variety of other disorders and mechanisms. A study by Yang et al. found abnormal endothelial function as measured by arterial flow-mediated vasodilation in patients with LV versus controls.^58^ In patients with sickle cell disease, LV may be related to endothelial cell injury or intrinsic hypercoagulability, with microtrauma serving as the trigger for ulceration.^59,60^ Whether hydroxyurea is beneficial in treating ulcers, or detrimental in causing them, is unclear and may be disease-specific. LV resolution following hydroxyurea initiation has been described in sickle cell disease; however, LV has also been described as a consequence of hydroxyurea use for myeloproliferative disorders.^60,61^ The mechanism of hydroxyurea-induced ulceration may be related to its promotion of megaloblastic, nondeformable red blood cells causing microcirculatory impairment. This effect may be mitigated in sickle cell disease by promotion of fetal hemoglobin and hydroxyurea has not consistently been shown to be associated with ulceration in sickle cell disease.^62,63^ Recurrence of LV lesions has been seen in association with COVID-19 infection, which may be due to a combination of pro-inflammatory changes and hypercoagulability.^64^ To our knowledge, de novo livedoid vasculopathy in association with COVID-19 has not been reported. Associations have been reported with pregnancy, hematologic malignancies, and solid organ carcinomas as well.^3^

## Treatment

**Figure 4.**
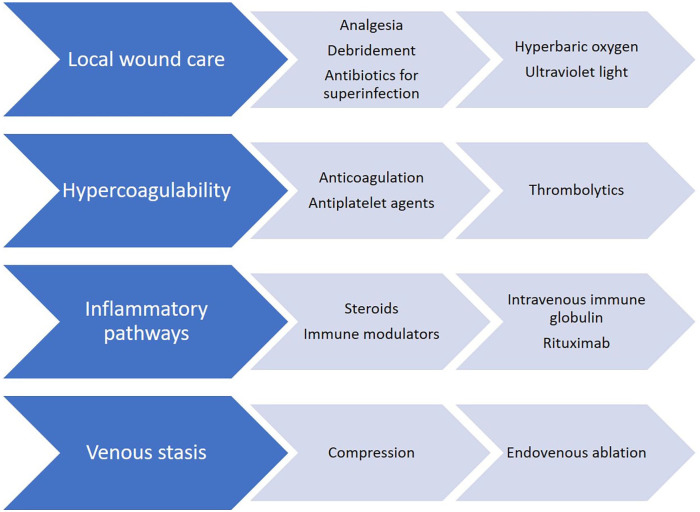
Treatment options for livedoid vasculopathy. Common office-based treatments are on the left, with treatments for special situations, such as refractory disease, on the right.

The incomplete understanding of the pathogenesis of LV is a major obstacle to effective treatment, and there are no targeted therapies for the condition. Additionally, the evidence base supporting LV therapies is limited to case reports and case series and there are no studies comparing the effectiveness of therapies, showing durable benefit of any single therapy, or demonstrating the capacity of therapy to prevent recurrences.^38^ Thus, management of LV relies heavily on empiric therapy, of which there are several options (Figure 4).

Oral anticoagulation is the most commonly reported treatment for LV and addresses dermal vessel thrombosis most directly.^38^ Of these, rivaroxaban is the most commonly used, and an uncontrolled phase 2a trial found a significant decrease in pain after 12 weeks of therapy.^65^ Antiplatelet agents, such as aspirin and pentoxifylline, are common alternatives. Low-dose systemic thrombolytics may be used in patients who are refractory to conventional therapies.^43^ Analgesia addressing ulcer-associated pain is essential and often the most immediate concern for patients.^14,66^ Local therapies for LV include regular wound debridement, hyperbaric oxygen, compression, and ultraviolet (UV) light. Of these, hyperbaric oxygen and compression therapy have been shown to promote fibrinolysis in addition to their respective effects in mitigating reperfusion injury and alleviating edema.^14,67^ Unfortunately, many insurers in the United States do not consider livedoid vasculopathy to be an approved indication for hyperbaric oxygen therapy, and the expense of this treatment is out of reach for many patients.^68^ Anabolic steroids and IVIG are among the handful of antiinflammatory agents found to be effective in LV, particularly in patients with associated connective tissue disease.^69,70^ The use of immunomodulatory agents such as TNF inhibitors has also been reported. In some patients, coexistent venous insufficiency will be identified and should be treated with compression therapy or venous intervention to eliminate venous hypertension and improve healing potential. Antibiotics may be necessary for superinfected ulcers.^71^ A freely downloadable patient information page on LV is include in this issue.^72^

## Summary

Livedoid vasculopathy presents significant challenges for patients and providers. The overlap in nomenclature and clinical appearance to other violaceous skin changes and ulcerative disorders is a source of confusion. The pathophysiology remains undetermined, and clarifying its etiology may be complicated by the grouping of patients with comorbidities varying from antiphospholipid syndrome to sickle cell disease under the umbrella of LV. Multiple factors may lead to microvascular thrombosis in LV. Hypercoagulability and immune dysregulation may predispose to thrombosis, with stasis and local endothelial dysfunction explaining the anatomic distribution of lesions, and trauma or inflammation possibly serving as triggers for ulcer formation.

Choosing appropriate treatment for a patient with LV is also difficult due to the wide variety of recommended and applied therapies, ranging from anticoagulation to steroids and endovascular ablation. This difficulty is compounded by the lack of good natural history studies and head-to-head or controlled trials evaluating specific treatments. Additionally, few studies are of sufficient duration to show lasting benefit of therapeutic interventions, which is particularly limiting in a disease that naturally remits and relapses.

**Figure 5.**
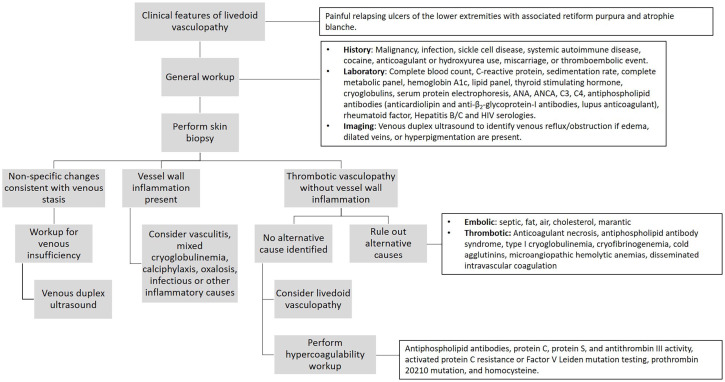
Diagnostic pathway for livedoid vasculopathy. ANA, antinuclear antibody; ANCA, antineutrophil cytoplasmic antibodies.

We suggest a standardized pathway for diagnosing livedoid vasculopathy outlined in Figure 5. We also recommend the use of terminology contextualizing LV by a patient’s clinical comorbidities (Table 2), with key points to remember summarized in Table 3. This may guide providers in ruling out conditions with similar terminology or appearance, evaluating for comorbidities, making referrals to appropriate specialists, and choosing from the available classes of therapy. A multidisciplinary and multiinstitutional approach to studying the natural history of livedoid vasculopathy is necessary to improve our understanding of the condition and advance therapeutic options. Finally, clarity in terminology is essential for diagnosis, patient education, and referral to the appropriate clinical and research resources.

**Table 2.** Proposed clinically focused, descriptive classification system for livedoid vasculopathy.

1 – Idiopathic livedoid vasculopathy
2 – Livedoid vasculopathy associated with hypercoagulable states
3 – Livedoid vasculopathy associated with rheumatic disease
4 – Livedoid vasculopathy with concurrent venous stasis
5 – Livedoid vasculopathy associated with other or multifactorial mechanisms

**Table 3.** Key points to remember about livedoid vasculopathy (LV).

• Clinical features of LV include recurrent, severely painful ulcers, violaceous skin discoloration, and atrophie blanche.
• LV is distinct from livedo reticularis, a benign skin discoloration without pain, ulceration, or clinical disease correlate.
• LV can be associated with hypercoagulable states, autoimmune disease, and venous stasis, but more than half of cases occur in individuals without medical comorbidity.
• Skin biopsy should always be performed to support LV diagnosis. Repeat biopsies may be necessary as findings are patchy and can be missed.
• Histologic features of LV include hyaline thickening of vessel walls in the papillary dermis with focal intraluminal thrombi.
• LV is a thrombotic vasculopathy distinct from vasculitis, and is not characterized by vessel wall damage or inflammatory infiltrate.
• The cause of LV is unknown, but it may be related to hypercoagulability, dysregulated inflammatory pathways, endothelial dysfunction, and venous stasis.
• Treatments for LV include analgesia, local wound care, antithrombotic therapy (anticoagulation and/or antiplatelet therapy), immune modulation, and treatment of concurrent venous stasis, if present.
